# Overproduction of α-Lipoic Acid by Gene Manipulated *Escherichia coli*

**DOI:** 10.1371/journal.pone.0169369

**Published:** 2017-01-09

**Authors:** Yirong Sun, Wenbin Zhang, Jincheng Ma, Hongshen Pang, Haihong Wang

**Affiliations:** 1 Guangzhou Institutes of Biomedicine and Health, Chinese Academy of Sciences, Guangzhou, Guangdong, P. R. China; 2 Guangdong Provincial Key Laboratory of Protein Function and Regulation in Agricultural Organisms, College of Life Sciences, South China Agricultural University, Guangzhou, Guangdong, P. R. China; 3 Shenzhen University, Shenzhen, Guangdong, P.R.China; Infectious Disease Research Institute, UNITED STATES

## Abstract

Alpha-lipoic acid (LA) is an important enzyme cofactor widely used by organisms and is also a natural antioxidant for the treatment of pathologies driven by low levels of endogenous antioxidants. In order to establish a safer and more efficient process for LA production, we developed a new biological method for LA synthesis based on the emerging knowledge of lipoic acid biosynthesis. We first cloned the *lipD* gene, which encodes the lipoyl domain of the E2 subunit of pyruvate dehydrogenase, allowing high levels of LipD production. Plasmids containing genes for the biosynthesis of LA were subsequently constructed utilizing various vectors and promotors to produce high levels of LA. These plasmids were transformed into the *Escherichia coli* strain BL21. Octanoic acid (OA) was used as the substrate for LA synthesis. One transformant, YS61, which carried *lipD*, *lplA*, and *lipA*, produced LA at levels over 200-fold greater than the wild-type strain, showing that LA could be produced efficiently in *E*. *coli* using genetic engineering methods.

## Introduction

LA is a sulfur-containing cofactor found in most prokaryotic and eukaryotic organisms. It exists in water soluble oxidized and reduced forms [1]. As an antioxidant, the beneficial effects of LA have been shown on three fronts: (1) scavenging free radicals and reactive oxygen species; (2) chelating metal ions; (3) the regeneration of other antioxidants, such as vitamin E, vitamin C and glutathione [2], which have applications in the prevention and treatment of diabetes (including chronic complications), cerebral and neuro-degenerative diseases, radiation injury, ischemia reperfusion injury, and AIDS [3–6]. Furthermore, it has recently been reported that LA alone or in combination with paclitaxel can inhibit NF-κB expression and thus, inhibit breast cancer cell proliferation [7].

LA is essential for the function of several key enzymes involved in oxidative and single carbon metabolism including pyruvate dehydrogenase (PDH), 2-oxoglutarate dehydrogenase (2-OGDH), branched-chain 2-oxo-acid dehydrogenase, acetoin dehydrogenase and the glycine cleavage system [8, 9]. In each enzyme, a specific subunit is modified by covalent attachment of LA to the ε-amino group of specific lysine residues within conserved domains (called lipoyl domain) of these subunits. The gene that encodes the lipoyl domain was named as *lipD* in this research. The protein-bound lipoamide moieties serve as carriers for reaction intermediates within the multiple active sites of these multi-enzyme complexes [10–13].

**Fig 1:**
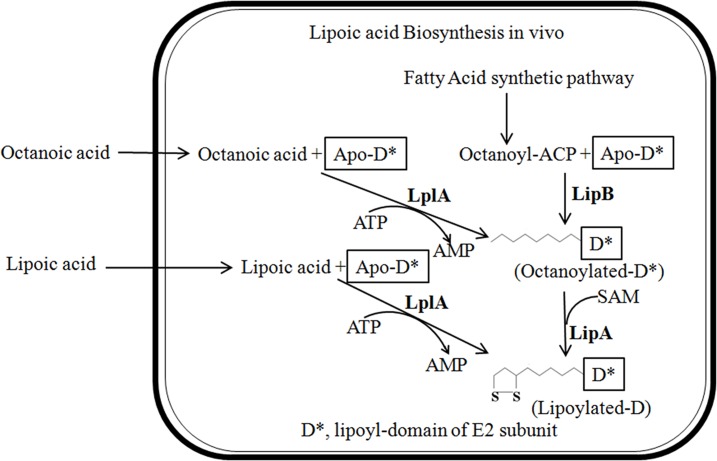
The pathway of lipoic acid synthesis in *E*. *coli*. The roundish rectangle represents an *E*. *coli* cell. Two complementary systems for protein lipoylation in *E*. *coli* are shown. Exogenous lipoic acid or octanoic acid is transferred to unlipoylated apo proteins in an ATP-dependent manner by LplA. The second *E*. *coli* pathway requires LipB to transfer endogenously synthesized octanoic acid to apo proteins, which then becomes the substrate for sulfur insertion by LipA. The carboxyl group of octanoic acid and lipoic acid is linked to the ε-amino group of a specific lysine residue of the lipoyl domain of the E2 subunit.

Although the functions of LA in multi-enzyme complexes and human health have been well studied over the past forty years, an understanding of the LA biosynthetic pathway has only recently been appreciated. The biosynthetic pathway was uncovered most clearly in *E*. *coli* over the past two decades, revealing that LA is synthesized *de novo* from an intermediate in fatty acid metabolism. As showed in Fig 1, besides the fact that the lipoyl domain was necessary for LA biosynthesis, three enzymes participate in LA synthesis in *E*. *coli*: LplA (lipoate-protein ligase), LipB (octanoyl protein ACP carrier protein: protein transferase), and LipA (lipoic acid synthase) [14]. LplA, encoded by the *lplA* gene [15], transfers exogenous LA to the unlipoylated-apo-lipoyl domain of the E2 subunit in an ATP-dependent manner [16], and can also catalyze the conjugation of exogenous octanoic acid and the target enzyme as shown in Fig 1 [17]. LipB, encoded by the *lipB* gene, catalyzes the transfer of an octanoyl residue from ACP to the target enzyme [18, 19]. LipA, encoded by the *lipA* gene, is responsible for the formation of two C-S bonds. The LipA-driven reaction requires iron-sulfur clusters (4Fe-4S) and SAM (produced by the *metK* gene) in order to perform its function [20–24].

Nowadays, LA is mainly produced by chemical synthesis and extraction from organic compounds. LA content from dietary sources is very low. Ten tons of liver contains only 30 mg of LA [25, 26]. Chemical synthesis of LA requires toxic catalysts to achieve addition of two sulfur atoms, which creates an environmental concern over increased pollution [27]. In addition, (R)-(+)-lipoic acid (RLA) exists in natural form in the human body and other organisms, while (S)-(-)-lipoic acid (SLA) has no function [28, 29]. LA synthesized chemically is composed of equal amounts of RLA and SLA, and separation of RLA from the mixture is difficult [27–29].

In this study, we combined the recently uncovered understanding of LA biosynthesis with genetic engineering strategies to construct *E*. *coli* strains that could yield improved production of LA. Four genes, *lipD*, *lplA*, *metK* and *lipA*, were cloned. Culture conditions for *E*. *coli* strains harboring these cloned genes were optimized. Strains constructed in this study could produce over 200-fold greater yield of LA under optimized culture conditions.

## Materials and Methods

### Bacterial strains, plasmids, and culture media

The *E*. *coli* strains and plasmids used in this study are listed in Table 1. LB medium was generally used, and 2YT was used as fermentation medium [30]. Cells were grown at 37°C. Antibiotics were used at the following concentrations: ampicillin, 100μg/mL; kanamycin, 30 μg/mL; chloramphenicol, 30 μg/mL. Isopropyl β-D-1-galactopyranoside (IPTG), L-arabinose, and standard LA were purchased from Sigma-Aldrich (St. Louis, Missouri, USA). The concentration of L-arabinose used was 1 mM. One molar OA solution was prepared by mixing 3.23 mL of OA (liquid), 2 mL of 10 N KOH, and 14.77 mL of alcohol. One mg/mL of LA solution was prepared by the addition of 10 mg of LA to 10 mL of ethanol. Trace metal mixture (1000×) contained 50 mM FeCl_3_, 20 mM CaCl_2_, 10 mM MnCl_2_ and ZnSO_4_, and 2 mM CoCl_2_, CuCl_2_, NiCl_2_, Na_2_MoO_4_, Na_2_SeO_3_, and H_3_BO_3_ [31]. Mutants were grown at 37°C in EG medium, which is minimal E medium containing 0.4% glucose [32, 33].

**Table 1:** Strains and plasmids.

Strains and plasmids	Relevant genotype or characteristics	Sources or reference
Strains		
DH5α	*lacZ*ΔM15,Δ(*lacZYA-argF*)*U169*,*recA1*,*endA1*, *hsdR17*	(Taylor,1993)
BL21(DE3)	F-, *dcm*, *omp*T, *hsd*S(r_b-_m_B-_), *gal*	(Studier, 1986)
TM179	*rpsL*,*lipA150*::*Tn1000*dKn,*lipB175Tn10*dTc	(Morris et al., 1995)
TM245	JK1/pGS331	(Morris et al., 1995)
MG1665	*F*^*-*^, λ^*-*^,*ilvG*, *rfb50*, *rph1*	(Taylor et al., 1993)
YS17	BL21(DE3)/pYS17	This work
YS18	BL21(DE3)/pYS18	This work
YS19	DH5α/pYS19	This work
YS25	BL21(DE3)/pYS25	This work
YS47	BL21(DE3)/pYS47	This work
YS40	TM136/pGSS331	This work
YS55	BL21(DE3)/pYS17&pYS1	This work
YS56	BL21(DE3)/pYS25&pYS27	This work
YS57	BL21(DE3)/pYS47&pYS49	This work
YS58	BL21(DE3)/pYS47&pYS48	This work
YS59	BL21(DE3)/pYS25&pYS48	This work
YS60	BL21(DE3)/pYS25&pYS49	This work
YS61	BL21(DE3)/pYS47&pYS27	This work
Plasmids		
pGS331	Amp^r^, p*tac*85 *lipD*	(Ali and Guest, 1990)
pSU18	Cm^r^, LacZa Rep(pl5A)	(Bolland et al., 1990)
pET15	Amp^r^, T7 expression plasmid	Novagen
pET28	Km^r^, T7 expression plasmid	Novagen
pBAD24	Amp^r^, *araC*-PBAD promoter	(Guzman L M, 1995)
pBAD34	Cm^r^, *araC*-PBAD promoter	(Guzman L M, 1995)
pMD19-T	Amp^r^, TA cloning vecor	Takara
pYS1	Cm^r^, pSU18 carrying *lplA*	This work
pYS17	Km^r^, pET28 carrying *lipD* from pGSS331	This work
pYS18	Amp^r^, pET15 carrying *lipD* from pGSS331	This work
pYS19	Amp^r^, pBAD24 carrying *lipD* from pGSS331	This work
pYS25	Km^r^, pET28 carrying *lipD-lpl*A	This work
pYS27	Cm^r^, pBAD34 carrying *lip*A	This work
pYS33	Amp^r^, pMD19-T carrying *met*K	This work
pYS47	Km^r^, pET28 carrying *lip*D*-*p*tac85-lpl*A	This work
pYS48	Cm^r^, pBAD34 carrying *lip*A*-sd*(*Nde*I)*-met*K	This work
pYS49	Cm^r^, pBAD34 carrying *lip*A*-*(*Xba*I)*-met*K	This work

Amp^r^, resistant to ampicillin; Km^r^, resistant to kanamycin; Cm^r^, resistant to chloramphenicol

### Construction of plasmids for overexpression of the lipoyl domain and lplA

The lipoyl domain should be produced at high levels since the biosynthesis of LA bound to it is required. Plasmid pGS331, which carries the lipoyl domain encoded gene (*lipD*) from *E*. *coli* [10], was donated by Prof. Cronan from the University of Illinois (Chigago, Illinois, USA). The *lipD* fragment from pGS331 was cloned into pET28 and pBAD24. Restriction enzyme digestion with *Nco*I and *Sal*I generated pYS17 and pYS19. The *lipD* fragment from pYS17 was inserted into the *Nco*I-*Xho*I sites of pET15 to obtain pYS18(Table 1).

In order to obtain the octanoylated-lipoyl domain from overexpression of the apo-lipoyl domain, the plasmids carrying *lipD* and *lplA* were constructed as follows. The fragment containing *lipD* was obtained by PCR amplification of pYS17 (Table 1) template DNA using primers 43 and 239 (Table 2). The *lplA* gene fragment from *E*.*coli* was amplified by PCR using pYS1 template DNA, which was yielded by the *lplA* gene cloned into the *Xba*I-*Sal*I restriction sites of pSU18 (Table 1), and also using primers 238 and 240 (Table 2). Purified PCR fragments containing both *lipD* and *lplA* were obtained by overlap PCR in which the two fragments were mixed at equal amounts along with primers 43 and 240 (Table 2). The resulting PCR fragment was cloned into vector pMD19-T (Takara, Otsu, shiga, Japan) and finally inserted into *Noc*I-*BamH*I sites of pET28 to obtain pYS25. BL21 (DE3) was transferred into plasmid pYS25 to obtain YS25 (Table 1).

**Table 2:** Primers.

No.	Sequence (5’-3’)
43	TAATACGACTCACTATAGGGG
77	GTAAGTAATTACTGCAGGATTAC
78	GAACACGCACGTCATGAGTAAAC
240	CTTGGATCCCTGCAGGTAACTACCTTACAGC
227	TCTAGACATATGGCAAAACACCT
228	GTCGACGGCCTTTGAACGCAG
238	GCAGCTCCTGCGTAACATATGTCCACATTACGC
239	GCGTAATGTGGACATATGTTACGCAGGAGCTGC
277	GAGCTGGATCCCATATGCGTTTCACTCCTCTAGATTACTTAACTTCCATCCCTTTCG
301	GAGCAGGCGTAATGTGGACATGGATCCTGTTTCCTG
302	GTCTATGAATTCACTCCCCATCCCCCTGT
303	CAGGAAACAGGATCCATGTCCACATTACGCCTGCTC

To control *lplA* expression, a plasmid with both *lipD* and *lplA* driven by different promoters from that of pYS25 was constructed. Primers 301, 302 and 303 were designed according to the p*tac85* promoter and *lplA* sequence (Table 2). The p*tac85*-*lplA* fragment which was obtained by overlap PCR, was cloned into the *EcoR*I-*Sal*I restriction sites of pYS17 (pET28-*lipD*) to yield plasmid pYS47. Plasmid pYS47 was transferred to BL21 (DE3) to obtain YS47 (Table 1).

### Construction of strains for overproduction of lipoic acid

It was necessary to catalyze the octanoylated-lipoyl domain to holo-lipoyl domain and supply enough sulfur atoms for this transformation. The *lipA* gene from *E*. *coli* was amplified using primers 77 and 78 (Table 2) and the resulting PCR fragment was cloned into *Pst*I-*BspH*I sites in the vector pBAD34 to obtain pYS27 (Table 2). The *metK* fragment of *E*. *coli* was obtained by PCR using primers 227 and 228 (Table 2) and subsequently cloned into vector pMD19-T to obtain pYS33. Primer 277, which contains the SD sequence from pBAD43 and three restriction enzyme sites (*Xba*I, *Nde*I and *BamH*I), was designed to obtain the *lipA*-*sd* fragment (Table 2). The fragment containing *lipA-sd* and *metK* from pYS33 was inserted into *Eco*I-*Nde*I and *Nde*I-*Sal*I sites of pBAD34 separately to yield pYS48 that carries the *lipA-sd-metK* fragment. The *lipA-sd* and *metK* fragment from pYS33 was cloned into *Eco*I-*Xba*I and *Xba*I-*Sal*I sites of pBAD34 separately to obtain pYS49 that carries fragment *lipA-metK* (Tables 1 and 2).

All genes constructed using PCR were sequenced, with results confirming correct sequences.

All genes required for LA biosynthesis were constructed in five different plasmids. For LA synthesis, we constructed six strains using the BL21 (DE3) parent strain: YS56 (carrying pYS25 and pYS27), YS57 (carrying pYS47 and pYS49), YS58 (carrying pYS47 and pYS48), YS59 (carrying pYS25 and pYS48), YS60 (carrying pYS25 and pYS49), and YS61 (carrying pYS47 and pYS27).

### Purification and assay of the lipoyl domain

The lipoyl domain was purified as described by Ali and Guest (1990). After the cells engineered to produce the lipoyl domain were pre-cultured overnight in LB medium with appropriate antibiotics, the cells were diluted 1,000-fold with fresh LB medium, induced for 3 hours after the concentration of cells at an OD_600_ of 0.3.The cells were harvested by centrifugation at 5,000 × g for 5 minutes at 4°C and then re-suspended in 20 mM sodium phosphate buffer, pH 7.0, containing EDTA (2 mM), NaN_3_ (0.02%), phenylmethanesulphonyl fluoride (1 mM) and benzamidine hydrochloride (1 mM). The cells were lysed by ultrasonic treatment at 4°C. The supernatant was obtained by centrifugation at 38,000 × g for 30 minutes, adjusted to pH 4 by the addition of 1 M HCl and was again centrifuged at 38,000 × g for 30 minutes. The lipoyl domain was purified by gel filtration using the Sephadex G-75 column after dialysis by 10 mM ammonium acetate at 4°C. The fractions containing the lipoyl domain were passed through a strong anion exchange resin (UNOsphere, Q, Bio-Rad, California, USA) with a gradient from 10 mM to 1 M ammonium acetate as a mobile phase.

Morris reported that the apo-lipoyl domain, octanoylated-lipoyl domain, and holo-lipoyl domain showed different velocities in non-denaturing gel electrophoresis [34]. The lipoyl domain was assayed by non-denaturing PAGE [stacking gel (pH 6.8) 50% T, 2.50% C; resolving gel (pH 8.3)20% T, 0.6% C], followed by quantitative densitometry of Coomassie Brilliant Blue-stained gels. Reference samples of the pure domain were applied to the gel [34, 35].

### LA extraction

Cells for producing lipoic acid were cultured for 6 hours and then for an additional 3 hours in the presence of 1 mM IPTG plus 1 mM OA. These cells from one liter of culture medium were harvested by centrifugation and subsequently were disrupted by ultrasonic treatment. LA was released from the lipoylated-lipoyl domain (holo-lipoyl domain) by hydrolysis with 3 M H_2_SO_4_ in a 200-mL round-bottom flask. After all lipoyl domains had been incubated at 120°C for 2 hours, the pH was adjusted to 7.0 with 4 N NaOH [25, 36]. The organic phase was separated after the addition of an equal volume of benzene. Pure LA was obtained by distillation of the organic solution. All steps were carried out in a ventilation cabinet. The crystals of standard LA were dissolved in 1 mL of ethanol and stored at –20°C.

### Determination of α-lipoic acid concentration

Biological method: LA concentration was assayed with *E*. *coli* mutant strain TM179 in which the *lipA* and *lipB* mutant cannot synthesize LA by itself, as described previously [25]. Standard LA was added in the range of 0.2 to 2.0 ng per culture medium. Purified LA prepared from the various strains were diluted in the above range and added to the culture medium. All strains were cultured at the same time, and the amounts of LA were estimated by the growth of the mutant *E*. *coli* strain in E-minimal medium with different concentration of LA after two days.

HPLC method: A mixture of 1 volume of KH_2_PO_4_ solution (pH 2, 25 mM) and three volumes of methanol were used as the mobile phase. The C18 column was purchased from Waters Company (Milford, Massachusetts, USA). The UV absorption was detected at wavelength 330 nm [37].The standard curve for the HPLC analysis was generated by the standard LA in a range from 10 ng to 100 μg.

LCMS method: Chromatographic separation by Agilent 1290 UPLC system (Agilent Technologies, Walbronn, Germany) was used to inject 5 μL samples on a 1.8 μM C18 column (50 × 2.1 mm). The mobile phase composed of 0.05 M formic acid- acetonitrile mixture (40:60, v/v) was delivered at 0.1 mL/min. The HPLC system was interfaced to an Agilent 6540B Q-TOF series MS system (Agilent Technologies, Walbronn, Germany), and mass spectra were acquired at a scan rate of 5.0 spectra/s with a mass range of 100–1100 M/z [38].

### Other methods

The protein concentration was determined by the Bradford assay using an absorption peak at 595 nm [39]. The reagents were purchased from Bio-Rad. Bovine serum albumin was used as the standard. The protein concentration in the gel was analyzed with BandScan5.0 protein analysis software. Plasmid DNA was isolated and manipulated by standard procedures (Sambrook, 1989). The dry weight of cells was calculated by first drying the cells by desiccation and calculating their weigh described previously [25].

## Results

### Comparing production of the lipoyl domain from different plasmids

**Fig 2:**
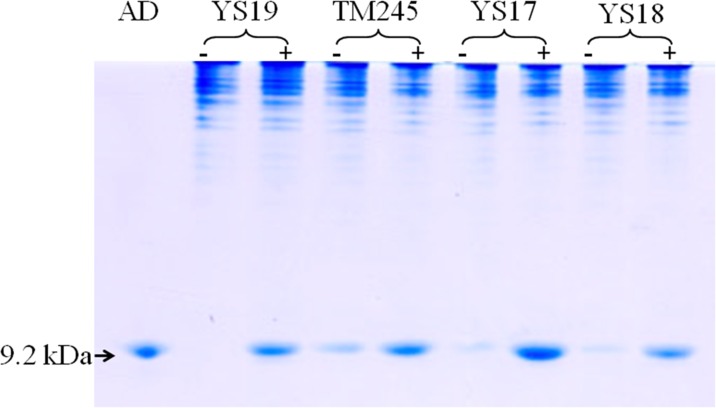
The expression of the apo-lipoyl domain in strains with various vectors. Indicated strains were cultured in LB medium to OD_600_ = 0.3, and the expression of the apo-lipoyl domain was induced for 3 hours. A dash (-) indicates that neither IPTG nor arabinose was added, while a plus sign (+) indicates IPTG (TM245, YS17 and YS28) or arabinose (YS19) was added for induction. The lipoyl domain was analyzed as described in the Materials and Methods section. The gel assay was conducted using non-denaturing polyacrylamide gel electrophoresis.

Since LA biosynthesis is closely linked with apo-lipoyl domain, this domain should be expressed at high levels in order to achieve large-scale production of LA. Several constructs were tested for yield of the apo-lipoyl domain, and the purified apo-lipoyl domain with the molecular weight of about 9.2 kD was used as the standard control. Yield from strains, YS19, TM245, YS17 and YS18 were calculated as 142.12±16.01, 202.23±24.11, 324.42±38.37, and 208.35±24.18 μg/mg dry weight of cells, respectively (Fig 2). Based on these results, pET28 was chosen as the construct for the subsequent study.

### Construction of plasmids carrying both *lplA* and *lipD*

**Fig 3:**
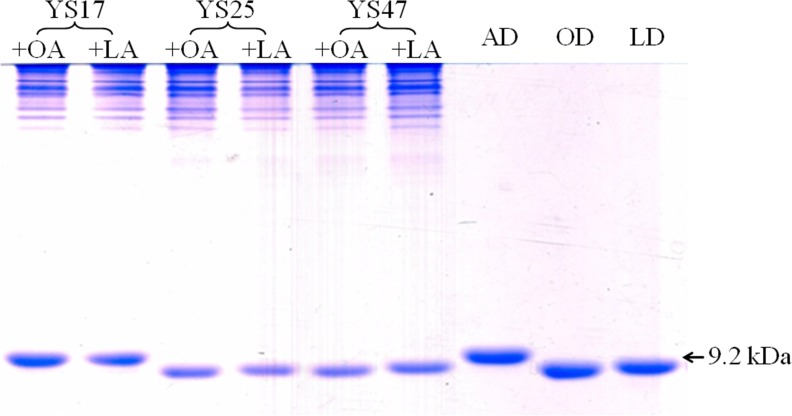
Production of apo-, octanoylated- and holo-lipoyl domains in strains with a plasmid carrying *lipD* and *lplA*. Indicated strains were cultured in LB medium and then cultured with OA or LA for 3 hours to produce the respective domains. The domains were analyzed as described in the Materials and Methods section. The gel shift assay was conducted using non-denaturing polyacrylamide gel electrophoresis. AD, apo-lipoyl domain; OD, octanoylated-lipoyl domain; LD, holo-lipoyl domain.

Low yield of the octanoylated-lipoyl domain and holo-lipoyl domain was obtained in YS17 even when OA or LA was added (Fig 3). This indicates that the LplA activity in YS17 is insufficient to produce the octanoylated- or holo-lipoyl domain. The activity of LplA needs to be increased with the aim of catalyzing the overproduction of the apo-lipoyl domain, thus driving production of the octanoylated-lipoyl domain.

The *lipD* and *lplA* genes were inserted into vector pET28 with either the same promoter or different promoters, which gave rise to YS25 and YS47 (Table 1). YS25 and YS47, carrying both *lipD* and *lplA*, could fully convert the apo-lipoyl domain to the octanoylated-lipoyl domain or the holo-lipoyl domain when OA or LA were added as substrate, respectively (Fig 3). YS55, carrying *lplA* in pSU18, also produced the octanoylated- and holo-lipoyl domains, although the yield was less than that in YS25 and YS47 (data not shown).

**Fig 4:**
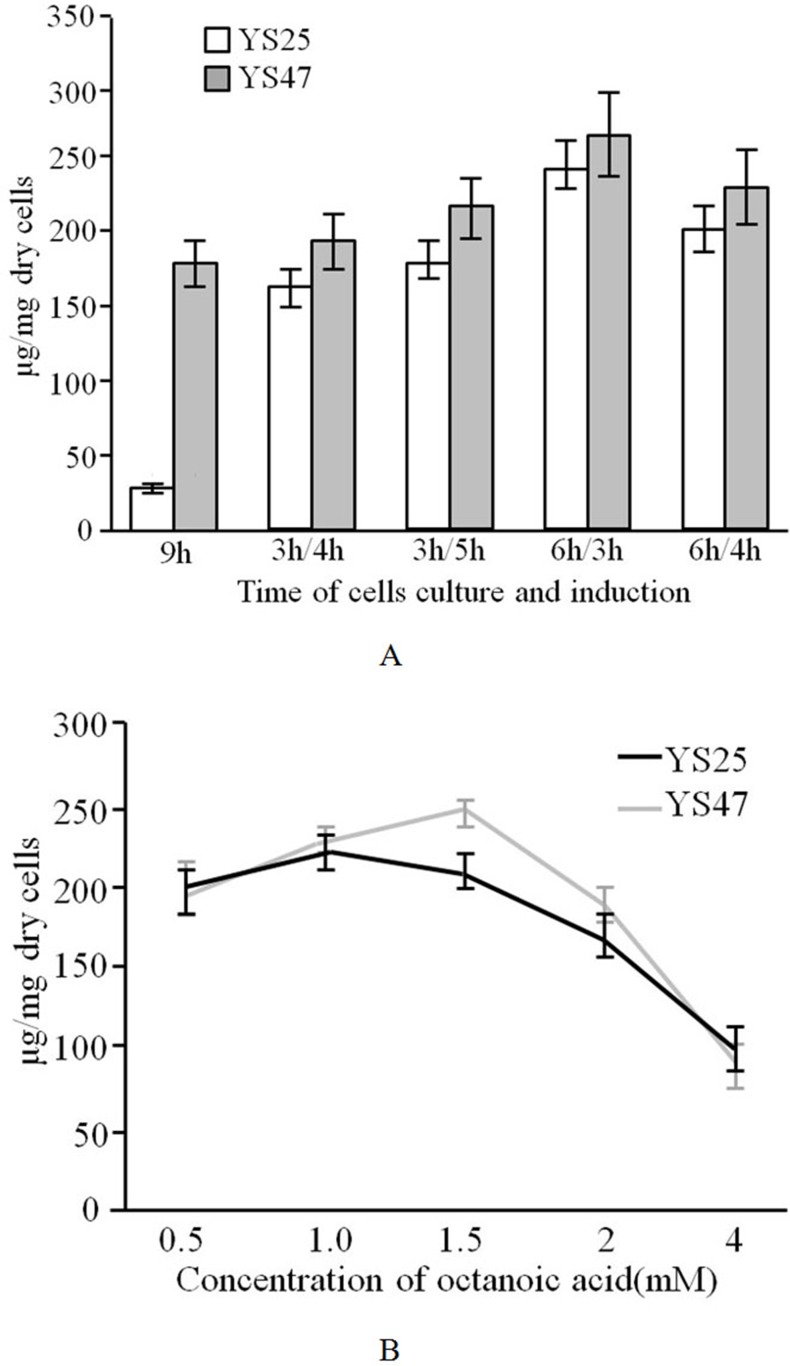
Octanoylated-lipoyl domain production in YS25 and YS47. (A) The production of the octanoylated-lipoyl domain under different culture conditions. ‘9h’ indicates 10 μM IPTG plus 1 mM OA were added, and cells were cultured for 9 hours; ‘3h/4h’ indicates cells were cultured for 3 hours and then 4 hours in the presence of 1 mM IPTG plus 1 mM OA; ‘3h/5h’ indicates cells were cultured for 3 hours and then 5 hours in the presence of 1 mM IPTG plus 1 mM OA; ‘6h/3h’ indicates cells were cultured for 6 hours and then 3 hours in the presence of 1 mM IPTG plus 1 mM OA; ‘6h/4h’ indicates cells were cultured for 6 hours and then 4 hours in the presence of 1 mM IPTG plus 1 mM OA. LB medium was used. (B) The production of the octanoylated-lipoyl domain in the presence of various concentrations of OA. YS25 and YS47 were cultured for 6 hours in LB medium and then 3 hours in the presence of 1 mM IPTG plus indicated concentrations of OA. Yield of the octanoylated—lipoyl domain were measured as described in Materials and Methods. Data from three independent experiments are expressed as mean ± S. D.

The production of the octanoylated-lipoyl domain in YS25 and YS47 was further examined under several different conditions for the induction of LipD and LplA: (1) 10 μM IPTG plus 1 mM OA were added, and cells were cultured for 9 hours. (2) Cells were cultured for 3 hours (OD_600_ was about 0.3) and then 4 or 5 hours in the presence of 1 mM IPTG plus 1 mM OA. (3) Cells were cultured for 6 hours (OD_600_ was about 0.8) and then 3 or 4 hours in the presence of 1 mM IPTG plus 1 mM OA. The highest yield of the octanoylated-lipoyl domain was obtained when YS25 and YS47 cells were cultured for 6 hours and induced for 3 hours (Fig 4A). YS47 produced a larger amount of the octanoylated-lipoyl domain than YS25.

We next measured the amount of octanoylated-lipoyl domain produced in YS25 and YS47 under different concentrations of OA (0.5 to 4 mM). YS25 and YS47 were cultured for 6 hours followed by additions of 1 mM IPTG plus OA (0.5, 1, 1.5, 2, and 4 mM). Cells were cultured for an additional 3 hours. The highest production of the octanoylated-lipoyl domain was observed with 1 and 1.5 mM OA in YS47 and YS25, respectively (Fig 4B). YS47 produced the octanoylated-lipoyl domain at a higher level than YS25 at concentrations of OA ranging from 1 to 2 mM. The results showed that the plasmid carrying both *lplA* with a *tac* promoter and *lipD* was useful for octanoylated-lipoyl domain production.

### Construction of transformants for producing the holo-lipoyl domain

Six transformants with combinations of five plasmids (pYS25, pYS47, pYS27, pYS48, and pYS49) were constructed. Plasmids pYS48 and pYS49 carried *lipA* with *metK*, while, pYS27 carried the single *lipA* gene. The expressions of LipA or LipA-metK were controlled by the *araBAD* promoter. The expression of LipD and LplA were controlled by the T7 promotor or tac promotor. Low levels of the holo-lipoyl domain were produced without the addition of L-arabinose in all transformants, while all strains produced the holo-lipoyl domain complement with the addition of L-arabinose and IPTG (data not shown).

**Fig 5:**
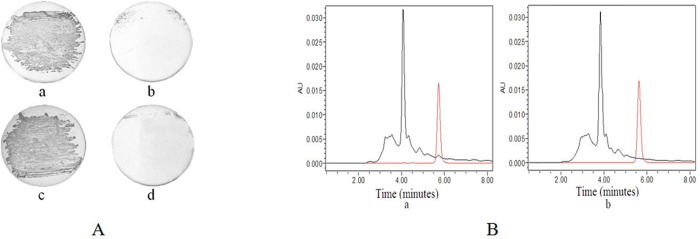
(A) Growth of TM179 in E minimal medium containing 1 μL of purified LA from YS56 (a), 1 μL of the purified LA from BL21 (b), 1 ng/mL of standard LA (c), or without any addition of LA (d). (B) The determination of LA concentration by HPLC. Ten microliters of the extracts from YS56 (a) and BL21 (b) were applied. Red lines were obtained with 7.5 μg of standard LA.

TM179 is a *lipB* and *lipA* double-deficient mutant of *E*. *coli* which requires LA for growth in E-minimal medium. When the extract from the strains described above were treated as described in the Materials and Methods section and the distillated sample was added to the medium, TM179 grew as well as in medium containing standard LA (Fig 5A). These results suggest that these strains can produce active LA. LA content produced by these strains ranged from 1.0 to 5.5 μg/mg dry weight of cells.

**Fig 6:**
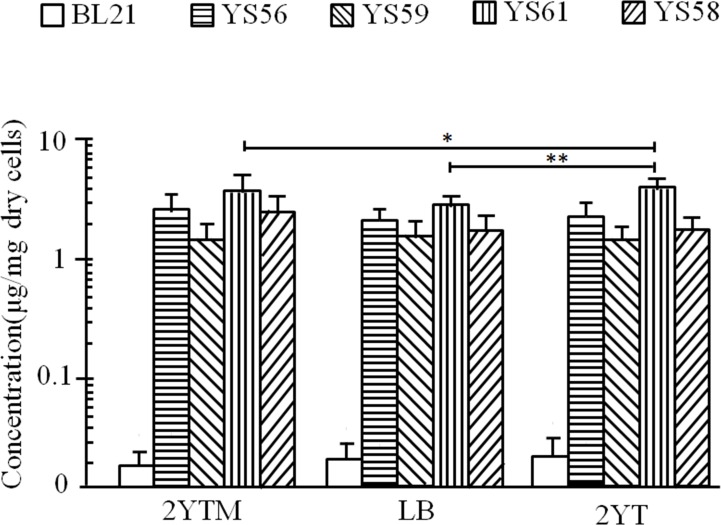
The production of LA in various media. Indicated strains were cultured for 6 hours and then 3 hours with 1 mM IPTG, 100 ng/mL L-arabinose and 1.5 mM OA. Media used were 2YTM (2YT plus trace metal mixture), LB and 2YT. The amounts of LA in the acid-treated extracts purified from these strains were assayed by HPLC. The averages ± standard deviations of the amount of lipoic acid obtained from three independent experiments and p values of the student t-test are represented. *, p<0.01(n = 6); **, p<0.005 (n = 6).

LA content in these strains was further measured using HPLC and LCMS. The absorption peak of standard LA was detected at 5.7 minutes (red curves in Fig 5B) in HPLC. The absorption peak of LA was detected in the sample prepared from YS56, but not BL21 (Fig 5B). These results were confirmed by the LCMS (S1 Fig). The content of LA was calculated as 3.90 ±0.75 μg/mg dry weight of YS56 cells (Fig 6, S1 Table). Similar results were obtained from YS58, YS59, and YS61, but the production with YS57 was poor. These results suggest that the constructed strains, especially YS56, YS58, YS59, and YS61, are useful for LA production.

### Optimization of culture medium conditions for LA production

Finally, the production of LA was examined with YS56, YS58, YS59, and YS61 in LB, 2YT, and 2YT plus 1× trace metal mixture (Studier, 2005). LA content was determined by HPLC (Fig 6, S1 Table). As shown in Fig 6, LA produced in YS61was greater in 2YT medium compared with other media. YS61 produced 5.25±0.43 μg/mg dry weight of cells in 2YT. LA production in the strain BL21 (DE3) and MG 1655 in 2YT medium was 0.020±0.011 μg/mg dry weight of cells. Thus, LA production in YS61 was more than 200-fold higher than that in the wild-type strain.

## Discussion

LA is mainly supplied industrially by chemical synthesis. There have been few studies aimed at scaling LA production biologically [40]. Currently, the production yield of LA by various microorganisms is too low for human use [25]. It has been reported that co-expression of LipA or LplA with a chaperone gene could increase LA production by 32%–110% compared with wild-type *E*. *coli* (about 0.090 μg/mg dry cell weight) [40]. In this study, we constructed *E*. *coli* strains YS56, YS58, YS59, and YS61 that could produce LA at greater than 200-fold higher levels than that in wild-type. In these strains, LipD, LplA, and LipA were co-expressed.

Biologically synthesized LA is bound to the lipoyl domain of α-keto carboxylic acid dehydrogenases, with no free LA existing in cells [41]. In order to produce LA at high levels, the lipoyl domain needs to be overproduced. The *lipD* gene that only produces the lipoly domain was cloned from various organisms and subsequently over-expressed in *E*. *coli* [11, 13]. In the present study, cloned *lipD* was used to produce the lipoyl domain at a higher level in *E*. *coli*. This may account for the high production of LA in our research.

It was surprising that production of LA was not increased by over-expression of MetK (Fig 6), which suggests that a sufficient S-adenosylmethionineis available without MetK over-expression. The synthesis of LA requires ATP as an energy source. It has been reported that cellular ATP content increases under acidic conditions [32, 42]. The production of LA decreased only slightly in acidic media of pH 5.5 to 6.5 (data not shown), suggesting that the enzymes synthesizing LA are still active under acidic conditions. The acidic medium could also be advantageous for fermentation of LA because microbial contamination could be minimized under these conditions.

A biological method for synthesis of LA has various advantages compared to chemical synthesis. The use of toxic catalysts is unavoidable in the chemical synthesis of LA. Toxic catalysts increase the risk to employees in plants and cause environmental pollution. Biological production has no such problems. Chemical synthesis produces a racemic mixture from which the separation of the (R)-enantiomer is difficult, while only active (R)-enantiomer is obtained by biosynthesis [28, 29], our results confirmed it too.

In conclusion, we constructed *E*. *coli* strains for the efficient biosynthesis of LA and established a preliminary fermentation method for producing LA. Future studies to optimize this method should also aim to reduce cost, thereby bringing cost in line with those associated with chemical synthesis.

## Supporting Information

S1 Fig The mass spectra Spectrum of five microliters of the extracts from YS61. (TIF)

S1 Table The production of LA in different strains and various media. (PDF)
