# Supplementary figures and images for: Overproduction of α-Lipoic Acid by Gene Manipulated Escherichia coli
Source: PLoS One. 2017 Jan 9;12(1):e0169369. doi: 10.1371/journal.pone.0169369 (PMC5222372; doi:10.1371/journal.pone.0169369)

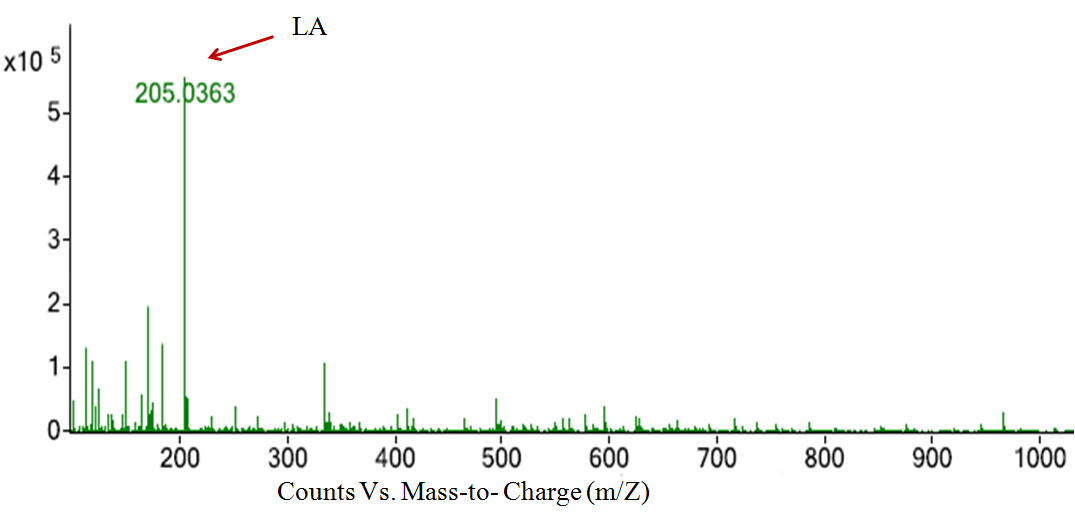

Supplement: S1 Fig — (TIF) [file pone.0169369.s001.tif]
