# Supplementary material for: Overproduction of α-Lipoic Acid by Gene Manipulated Escherichia coli
Source: PLoS One. 2017 Jan 9;12(1):e0169369. doi: 10.1371/journal.pone.0169369 (PMC5222372; doi:10.1371/journal.pone.0169369)
Supplement: S1 Table — (PDF) [file pone.0169369.s002.pdf]

S1 Talbe. **The production of LA in different strains and various media.** Indicated strains were cultured for 6 hours and then 3 hours with 1 mM IPTG, 100 ng/mL L-arabinose and 1.5 mM OA. Media used were 2YTM (2YT plus trace metal mixture), LB and 2YT. The amounts of LA in the acid-treated extracts purified from these strains were assayed by HPLC. The averages ± standard deviations of the amount of lipoic acid obtained from three independent experiments. Unit: μg/mg.

Strains \ media	2YTM	LB	2YT
BL21	0.017±0.003	0.019±0.004	0.020±0.011
YS56	3.68±0.66	3.80±0.89	3.90±0.75
YS59	2.08±0.61	3.22±0.53	2.65±0.50
YS61	4.50±0.36	4.20±0.47	5.25±0.43
YS58	3.40±0.58	3.34±0.65	3.60±0.62
